# Ductal Flow Ratio as Measure of Transition in Preterm Infants After Birth: A Pilot Study

**DOI:** 10.3389/fped.2021.668744

**Published:** 2021-07-19

**Authors:** Emma Brouwer, Ronny Knol, Nathan D. Hahurij, Stuart B. Hooper, Arjan B. Te Pas, Arno A. W. Roest

**Affiliations:** ^1^Division of Neonatology, Department of Paediatrics, Leiden University Medical Centre, Leiden, Netherlands; ^2^Division of Neonatology, Department of Paediatrics, Erasmus University Medical Centre, Rotterdam, Netherlands; ^3^Division of Paediatric Cardiology, Department of Paediatrics, Leiden University Medical Centre, Leiden, Netherlands; ^4^The Ritchie Centre, Hudson Institute of Medical Research, Monash University, Clayton, VIC, Australia

**Keywords:** preterm birth, ductus arteriosus, umbilical cord clamping, echocardiogaphy, neonatal transition

## Abstract

**Background:** Cardiovascular changes during the transition from intra- to extrauterine life, alters the pressure gradient across the ductus arteriosus (DA). DA flow ratio (R-L/L-R) has been suggested to reflect the infant's transitional status and could potentially predict neonatal outcomes after preterm birth.

**Aim:** Determine whether DA flow ratio correlates with oxygenation parameters in preterm infants at 1 h after birth.

**Methods:** Echocardiography was performed in preterm infants born <32 weeks gestational age (GA), as part of an ancillary study. DA flow was measured at 1 h after birth. DA flow ratio was correlated with FiO_2_, SpO_2_, and SpO_2_/FiO_2_ (SF) ratio. The DA flow ratio of infants receiving physiological-based cord clamping (PBCC) or time-based cord clamping (TBCC) were compared.

**Results:** Measurements from 16 infants were analysed (median [IQR] GA 29 [27–30] weeks; birthweight 1,176 [951–1,409] grams). R-L DA shunting was 16 [17–27] ml/kg/min and L-R was 110 [81–124] ml/kg/min. The DA flow ratio was 0.18 [0.11–0.28], SpO_2_ 94 [93–96]%, FiO_2_ was 23 [21–28]% and SF ratio 4.1 [3.3–4.5]. There was a moderate correlation between DA flow ratio and SpO_2_ [correlation coefficient (CC) −0.415; *p* = 0.110], FiO_2_ (CC 0.384; *p* = 0.142) and SF ratio (CC −0.356; *p* = 0.175). There were no differences in DA flow measurements between infants where PBBC or TBCC was performed.

**Conclusion:** In this pilot study we observed a non-significant positive correlation between DA flow ratio at 1 h after birth and oxygenation parameters in preterm infants.

## Introduction

Directly after birth, major cardiovascular changes occur during transition from intra-uterine to extra-uterine life. These cardiovascular changes are triggered by lung aeration and umbilical cord clamping. Lung aeration causes a decrease in pulmonary vascular resistance (PVR) and a corresponding increase in pulmonary blood flow (1, 2). In addition, after umbilical cord clamping, the systemic vascular resistance (SVR) increases following the loss of the low resistance placental circulation (3). The pressure changes in both the pulmonary and systemic circulation that accompany transition are reflected in the ductus arteriosus (DA) flow.

Both clinical and preclinical studies have evaluated DA flow to entirely flow from right-to-left (R-L) prior to birth, due a high PVR. During this time DA flow continues to be from R-L throughout diastole due to the retrograde flow in the pulmonary arteries (4, 5). As PVR decreases with lung aeration after birth, DA flow shifts from R-L flow to predominantly left-to-right (L-R) flow in the first 10 min after birth (4–6). It was suggested that echocardiographic measurements of DA flow ratio (R-L flow/ L-R flow) can be used as a measure of neonatal transitional, as duration, direction and the amount of DA flow are taken into account in this measure as well as pressure changes in the systemic circulation (4). DA flow ratio is likely to be influenced by the increase in SVR after cord clamping as well as the degree of lung aeration and decrease in PVR (4). As such, this measurement could theoretically be used to indicate a successful or disturbed neonatal transition, and even potentially predict (adverse) neonatal outcomes. However, data on these transitional changes in preterm infants is lacking.

Preterm born infants often fail to aerate their immature lungs and need respiratory support in order to transition from a foetus into a neonate. The success of transition is usually reflected by heart rate and pulmonary gas exchange efficiency, which is largely determined by the gas exchange surface area of the lungs and the gas diffusion distance, and is reflected by the need for altering the alveolar oxygen gradient (7, 8). Currently, there are various versions of an oxygenation index (OI) used to identify hypoxic respiratory failure in neonates and paediatric patients (9–11). We recently used the SpO_2_/FiO_2_ (SF) ratio as OI to reflect the gas exchange potential after birth (8). The SF ratio represents the gas exchange efficiency of the lungs and, next to lung immaturity, is largely influenced by the transitional status of the infant, particularly the degree of lung aeration (12). In this study, we aimed to investigate whether DA flow ratio is correlated with oxygenation parameters in preterm infants 1 h after birth as a measure of the success of neonatal transitional.

Experimental and clinical studies showed that infants went through transition more successfully when physiological based cord clamping (PBCC) was performed (13–15). As this observational study was performed while infants were recruited for an RCT comparing PBBC to time-based cord clamping (TBCC), we were able to compare the DA flow ratio between both groups (16).

## Methods

This pilot study is an ancillary study to the Aeration Breathing and Clamping (ABC) 2 and 3, multicentre randomised controlled trials (NTR7194; NCT03808051) performed in the Leiden University Medical Centre (LUMC). The ABC trials compared PBCC to TBCC on both short and long term outcomes in infants born <32 weeks gestation (16). This study was approved by the Institutional Review Board of the LUMC.

### Measurements

**Figure 1:**
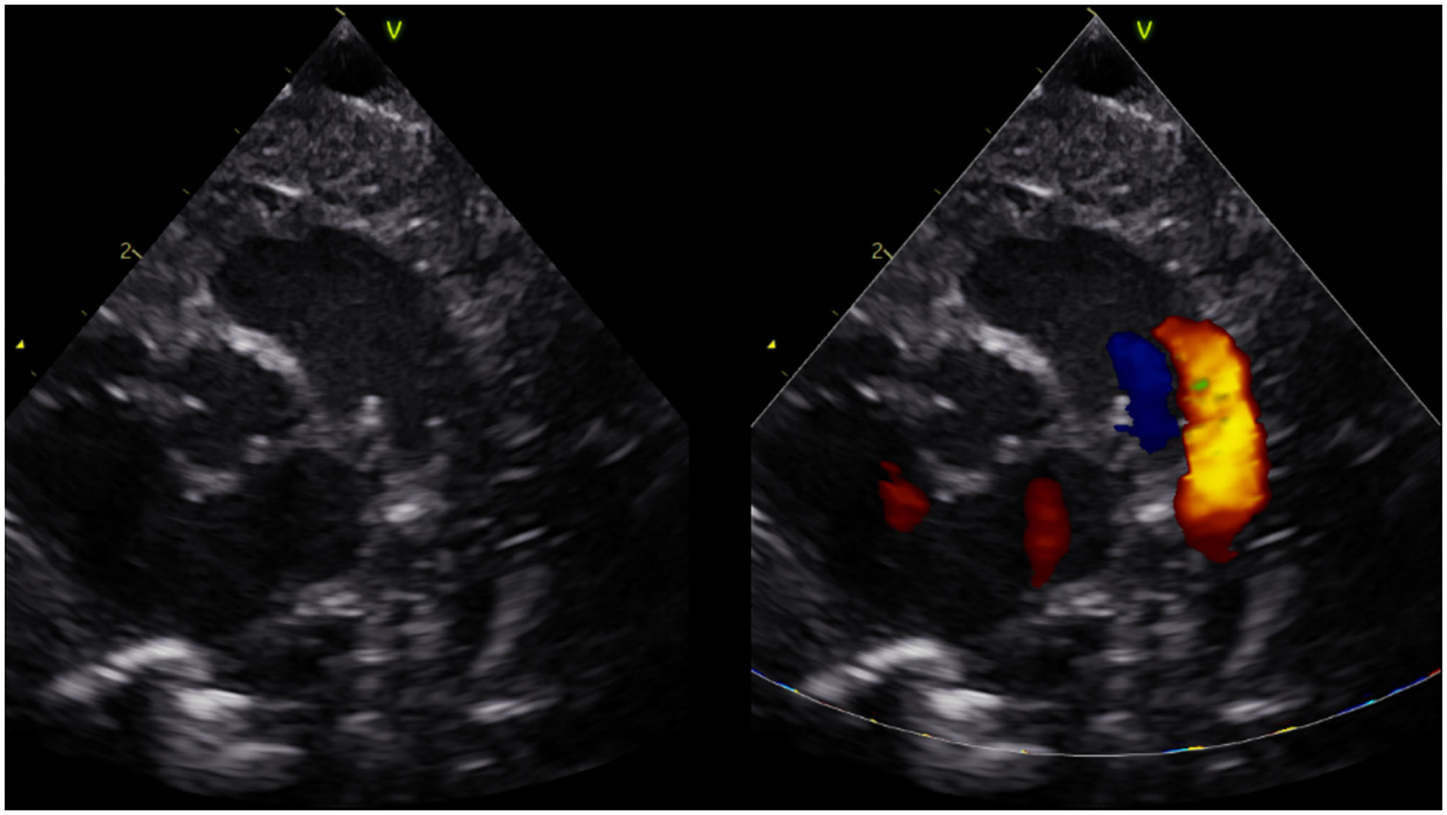
Short axis parasternal view of ductus arteriosus and pulmonary arteries (three vessel view).

**Figure 2:**
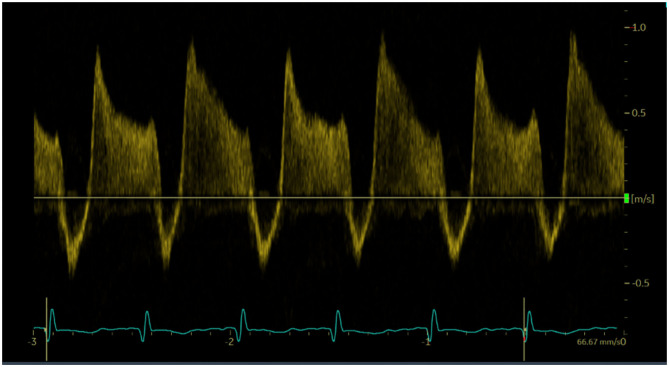
Doppler pulse wave measurements of ductus arteriosus flow. Three consecutive flows profiles were used to average measurements.

To assess DA flow and DA flow ratio, an echocardiographic examination was performed using a Vivid-S6 or Vivid-S60 (GE Healthcare) ultrasound system with a neonatal/paediatric 12S probe (4.0-11.0 MHz) 1 h after birth. Standard two-dimensional grey-scale images were acquired from the parasternal view and stored in digital format. The DA was visualised in the parasternal short axis view (Figure 1). To assess velocity time integral (VTI), pulsed wave Doppler measurements were obtained. Using pulsed wave Doppler measurements, the peak velocity of the R-L and L-R shunt was assessed (Figure 2). VTI evaluation of DA flow included analysis of three consecutive flow profiles, providing a mean VTI for each infant. Variation between the three consecutive flow profiles was calculated using standard deviation. DA shunt volume was calculated using the formula (((π × *Annulus*/4) × *HR* × *VTI*)/*birthweight*) (4). The net difference in DA shunting was calculated (L-R flow – R-L flow) as well as the DA flow ratio (R-L flow VTI/L-R flow VTI) to assess relative differences between R-L vs. L-R shunting, for each infant (4).

Measurements of SpO_2_ and FiO_2_ were averaged over a period of 5 min directly prior to the echocardiographic examination and SF ratio was calculated subsequently. The correlation between DA flow ratio and FiO_2_, SpO_2_, and SF ratio was calculated (SpO_2_/FiO_2_; maximum is 4.8; SpO_2_ 100%/FiO_2_ 21%) as well as the correlation between systolic blood pressure and DA flow ratio.

For this pilot study a convenience sample was used. The enrolment was based on the availability of the researcher and whether the echocardiographic ultrasound measurements would not interfere with essential neonatal care.

### Statistics

Data were analysed using SPSS software version 24.0 (SPSS IBM, Chicago, Illinois, USA). Results are presented as mean (standard deviation; SD), median [interquartile range; IQR] and *n* (%). Continuous data were analysed using the sample *t*-test or Mann-Whitney *U*-test, if appropriate. Correlation between DA ratio and oxygenation parameters was determined using the Spearman rank correlation test. A two-sided *p*-value of < 0.05 was regarded as statistically significant.

## Results

Echocardiographic measurements were performed in 21 out of 47 infants in the period from June 2018 until November 2018 and from October 2019 until September 2020. Ultrasound measurements were not made in a total of 26 infants; in 13 infants the researcher was unavailable for measurements and in 13 infants measurements would have interfered with needed neonatal care, which was determined by the caregiver and based on the specific treatment plan for each individual infant. In 5/21 infants, DA flow could not be successfully visualised, and therefore analysed, over a full cardiac cycle and measurements were therefore excluded, thus measurements from 16 infants (GA 29 [27–30] weeks; birthweight 1,176 [951–1,409] grams) were analysed (Table 1). Echocardiographic measurements were obtained at a median time of 01:13:00 [01:07:00–01:24:00] h after birth. One infant did not receive respiratory support during measurements, while all other infants received CPAP 7-8 cmH_2_O with automatic FiO_2_ control (SpO2 target ranges 91–95%). None of the infants received surfactant, fluid boluses or inotropics prior to ultrasound measurements. SpO_2_ and FiO_2_ during measurements were 94 [93–96]% and 0.23 [0.21–0.28]% respectively, with an SF ratio of 4.1 [3.3–4.5] (Table 2). Needed respiratory support in the first 24 h after birth, and need for surfactant of cardiovascular support in the first 48 h is summarised in Table 3.

**Table 1:** Baseline characteristics, *n* = 16.

Gestational age, weeks	29 [27–30]
Birthweight, grams	1176 [951–1,409]
Female	11 (68.8)
Antenatal corticosteroids	
- Yes, complete course - Yes, incomplete course - No	11 (68.8) 5 (31.3) 0 (0)
Caesarean section	7 (43.8)
Apgar 1 min	7 [4–8]
Apgar 5 min	8 [8–9]
Apgar 10 min	9 [9–10]
Respiratory support in delivery room	
- None - CPAP - PPV - Intubation	0 (0) 16 (100) 7 (43.8) 0 (0)
FiO_2_ max	0.85 [0.50–1.0]

*Values are presented as median [IQR] and n (%)*.

*CPAP, Continuous positive airway pressure; PPV, positive pressure ventilation*.

**Table 2:** Neonatal variables during echocardiographic measurements, *n* = 16.

Respiratory support	
- None - CPAP	1 (6) 15 (94)
SpO_2_	94 [93–96]
FiO_2_	0.23 [0.21–0.28]
Heartrate	152 [136–160]
Blood pressure	
- Systolic - Diastolic - MAP	52 [47–58] 34 [26–41] 41 [34–49]
First neonatal blood gas	
- pH - lactate	7.24 [7.16–7.30] 2.2 [1.6–6.1]

*Values are presented as n (%) and median [IQR]*.

*CPAP, Continuous positive airway pressure; MAP, Mean arterial blood pressure*.

**Table 3:** Respiratory and cardiovascular variables during the first 24 h after birth, *n* = 16.

Respiratory support 6 h	
- None - CPAP	1 (6) 15 (94)
PEEP	7 [6–8]
FiO_2_	0.22 [0.21–0.25]
Respiratory support 12h	
- None - CPAP	1 (6) 15 (94)
PEEP	7 [6–7]
FiO_2_	0.22 [0.21–0.25]
Respiratory support 24 h	
- None - High flow - CPAP	1 (6) 1 (6) 14 (88)
PEEP	7 (6–7)
FiO_2_	0.22 (0.21–0.25)
Need for surfactant <48 h	
- Yes - No	2 (12) 14 (88)
Cardiovascular support <48 h	
- None - Fluid bolus - Inotropics	14 (88) 2 (12) 0 (0)

*Values are presented as n (%) and median [IQR]*.

*CPAP, Continuous positive airway pressure; PEEP, Positive end expiratory pressure; FiO2, fractional inspired oxygen*.

**Figure 3:**
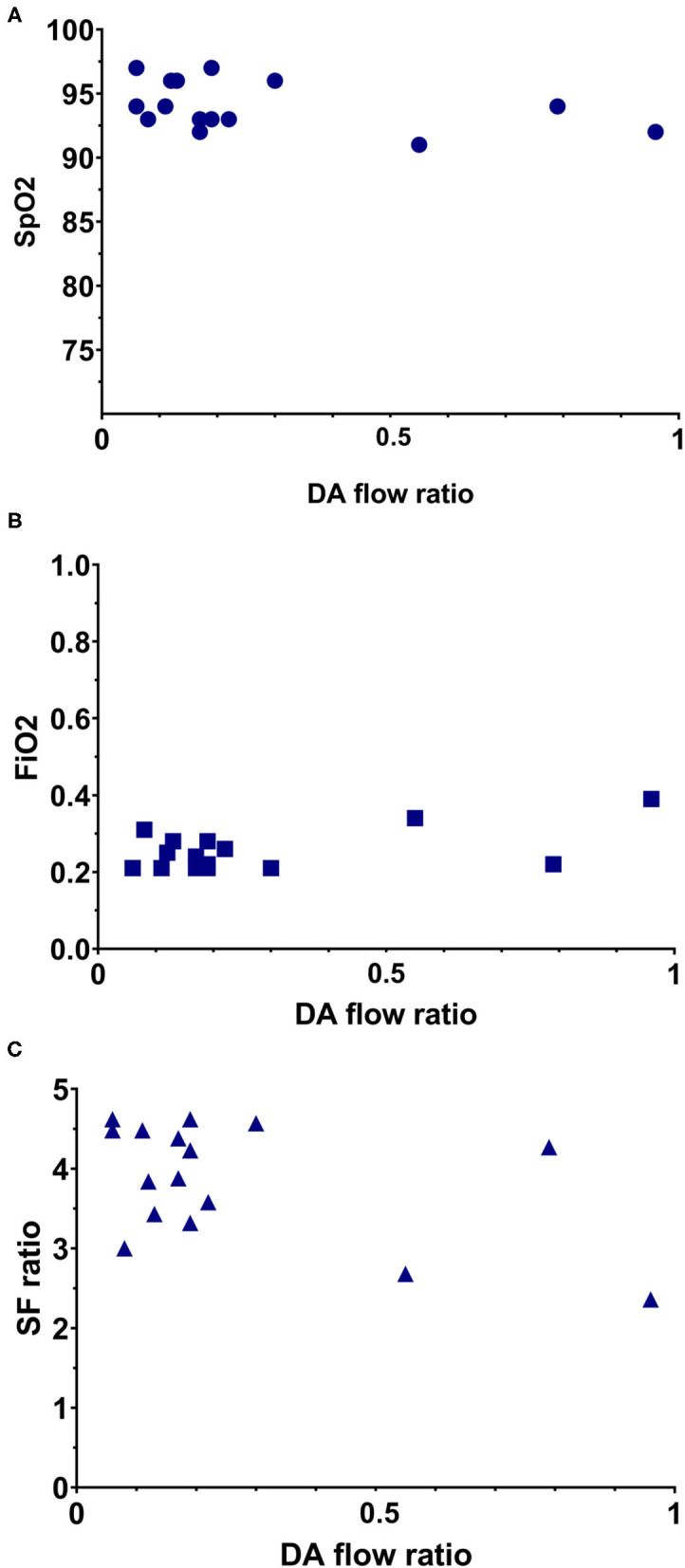
Simple scatterplot of the relation between DA flow ratio with oxygenation parameters. **(A)** Relation between DA flow ratio and the SpO_2_. **(B)** Relation between DA flow ratio and FiO_2_. **(C)** Relation between DA flow ratio and SF ratio.

Median R-L DA shunting was 16 [17–27] ml/kg/min, with a peak velocity of 0.52 [0.41–0.61] m/s and a mean VTI of 3.4 (1.6). Intra-patient SD for R-L VTI was 0.66 [0.39–1.03]. L-R shunting was 110 [81–124] ml/kg/min, with a peak velocity 0.83 [0.65–1.08] m/s and a VTI of 18.7 (8.4). Intra-patient SD for L-R VTI was 1.53 [1.23–3.67]. The net difference in DA shunting (L-R flow—R-L flow) was 89 [56–109] ml/kg/min. The DA flow ratio was 0.18 [0.11–0.28]. There was a moderate positive correlation between DA flow ratio and FiO_2_ (correlation coefficient (CC) 0.384; *p* = 0.142). There was a moderate negative correlation between DA flow ratio and SpO_2_ (CC −0.415; *p* = 0.110), SF ratio (CC −0.356; *p* = 0.175), and systolic pressure (CC −0.259; *p* = 0.300), however correlations remained non-significant (Figure 3). DA flow measurements and ratio where compared between infants who received PBCC or TBCC. One infant was excluded from this analysis based on an increased risk for pulmonary hypertension, based on rupture of membranes nearly 2 weeks prior to birth. Thus, 15 infants were compared (8 PBCC vs. 7 TBCC). We found no differences between groups for either R-L shunting (13 [8–44] vs. 17 [14–23] ml/kg/min, *p* = 0.30), L-R shunting (113 [86–122] vs. 97 [77–130] ml/kg/min, *p* = 0.82) or net difference in DA shunting (20 [7–23] vs. 14 [10–19], *p* = 0.42). No differences were found DA flow ratio between groups (0.12 [0.07–0.46] vs. 0.19 [0.17–0.22], *p* = 0.23).

## Discussion

In this pilot study we evaluated, for the first time, DA flow measurements in preterm infants at 1 h after birth. In this study we observed a bidirectional flow over the DA with a predominance of left-to-right shunting. While, the correlation between DA flow ratio and the SpO_2_, FiO_2_ and SF ratio were not significant, the tendency towards significance (*p*-values < 0.18) indicates that further investigation with a larger group of infants is required to determine whether the DA flow ratio has utility in indicating a successful or disturbed neonatal transition. Moreover, if DA flow ratio can successfully indicate a disturbed transition, based on the differential pressure changes in the pulmonary and systemic circulation, this measure can potentially predict underlying neonatal disease and (adverse) neonatal outcome.

We were able to successfully measure DA flow in 16 preterm infants at 1 h after birth and demonstrated DA flow to be predominantly L-R, indicated by the shunting volumes as well as the small DA flow ratio. Our findings are difficult to compare with the current literature as echocardiographic measurements in preterm infants after birth have previously focused on the presence of DA patency and not on DA flow changes after birth (17, 18). However, we previously demonstrated that in term born infants the DA flow shifts from R-L to predominantly L-R in the first minutes after birth (4) and remains bi-directional in the majority of infants on the first day of life. Similarly, we have now shown that preterm infants follow a similar temporal pattern (19). After birth, spontaneous breathing or assisted ventilation aerates the lung and thereby decreases PVR and increases pulmonary blood flow. This, combined with the increase in SVR that follows umbilical cord clamping, changes the pressure gradient across the DA and causes DA flow to reverse from R-L to predominantly L-R (20–22). The cardiac cycle dependent bidirectional flow pattern within the DA is thought to be due to the time differences between when the pressure waves generated by ventricular contraction reach each end of the DA (12). Early during systole the pressure wave emanating from the right ventricle reaches the pulmonary artery end of the DA first, resulting in R-L flow (5, 12). Then as the pressure way generated by the left ventricle reaches the aortic end of the DA, the pressure gradient across the DA reverses and the flow becomes L-R (5, 12). As such, measuring DA flow after birth, particularly during diastole, provides a direct indication of the pressure gradient across the DA and is highly indicative of the PVR relative to SVR.

Based on the correlation coefficient DA flow ratio showed a moderate negative correlation with both SpO_2_ and SF ratio and a moderate positive correlation with FiO_2_, however correlations remained non-significant. The negative correlation between SpO_2_ and SF ratio and DA ratio were expected, as good oxygenation with little use of additional oxygen would indicate a successful transition, and therefore low DA ratio's. Similarly, the positive correlation with FiO_2_ was expected, as high levels of additional oxygen would indicate a less successful transition, and therefore higher DA ratio's. All three oxygenation parameters are influenced by the changes during transition to extra-uterine life. After birth the primary site of gas exchange shifts to the lungs, after which oxygenation is largely determined by the surface area available for gas exchange, the gas diffusion distance and the partial pressure gradient for oxygen between the alveoli and adjacent capillaries. Thus, oxygenation depends on adequate lung aeration (surface area), gas diffusion distance and the amount of additional oxygen given during neonatal support (partial pressure gradient), all of which are largely dependent on the success of neonatal transition especially in preterm infants (7, 23).

The association between neonatal oxygenation and PVR has been demonstrated, with a reduced risk for an elevated PVR in preterm born infants with higher oxygenation targets (90–95%) (24). As both PVR and SVR are the main determinants of the DA pressure gradient, changes in PVR based on oxygenation status would subsequently result in changes in DA flow. In addition, studies investigating various oxygenation index's (such as SF ratio) demonstrated that SpO_2_ is moderately correlated to PVR in lambs (25). Nevertheless, while these oxygenation parameters can provide an indication of PVR, measuring DA flow using echocardiography would present a more direct and possibly a more realistic evaluation of PVR. If this is correct, this could explain why we observed moderate correlations between the oxygenation parameters and the DA flow ratio. As this was a pilot study with a small sample size and a relatively homogenous cohort, the correlations we observed were not statistically significant.

There are various factors that could influence DA shunting, i.e., changes in pulmonary pressure and haemodynamics, which could have had an effect on the obtained DA flow measurements. Infants in this study however, all received similar respiratory support, decreasing the possible influence of pressure change on our measurements. Changes in pulmonary venous return, heart rate, cardiac output and peripheral vascular resistance are all linked to either PVR or SVR, reflecting the neonatal transition, and will therefore be represented in the DA flow ratio.

Previous studies have already demonstrated that only 30-60 s of DCC has a positive effect on blood pressure and the need for inotropics in preterm infants in the first hours after birth (26–28). As PBCC improves hemodynamic stability during neonatal transition (15, 29), it is very well possible that this will further improves blood pressure for these infants. Therefore, DA flow could potentially demonstrate a more predominant L-R flow when compared to infants who received TBCC. However, the aim of this study was to investigate if DA flow ratio was correlated with oxygenation parameters and not to observe differences between groups who received different cord clamping strategies.

This study has some important limitations. The convenience sample used for this study was small, which may have had a limiting effect on the correlations. In addition, sample size was too small to find a significant difference in DA flow ratio. However, the results from this study are important as they could be used to calculate an appropriate sample size to demonstrate differences between groups in future studies. In a considerable number of infants who were eligible for this study echocardiographic measurement could not be obtained as the time point was not convenient and intervened with the clinical care, or the researcher was not available. When DA flow ratio were to be further assessed in a larger trial, echocardiographic measurements at different moments after birth would be recommended as well as having measurements performed by caregivers themselves.

In conclusion, this is the first study to report ultrasound measurements of DA flow at 1 h after birth in infants born <32 weeks gestational age. No differences were found when comparing measurements of infants who received PBCC and TBCC. We observed a non-significant positive correlation between the DA flow ratio and oxygenation parameters in preterm infants. Whether this parameter reflects the state of neonatal transition remains to be further investigated.

## Data Availability Statement

The raw data supporting the conclusions of this article will be made available by the authors, upon reasonable request.

## Ethics Statement

The studies involving human participants were reviewed and approved by Medical Research Ethics Committee of Leiden-Den Haag-Delft, Leiden University Medical Center. Written informed consent to participate in this study was provided by the participants' legal guardian/next of kin.

## Author Contributions

EB, RK, SH, AT, and AR wrote the ethics application. EB, AT, and AR participated in the study design and coordination, collected and analysed the data, and reviewed the literature. NH and SB participated in the study design. EB wrote the first draft and submitted the article. All authors participated in reviewing the data and editing the manuscript and read and approved the final manuscript.

## Conflict of Interest

The authors declare that the research was conducted in the absence of any commercial or financial relationships that could be construed as a potential conflict of interest.

## Abbreviations

DA: Ductus arteriosus
Rtol: right to left
LtoR: left to right
SF: SpO_2_/FiO_2_
PBCC: physiological-based cord clamping
TBCC: time-based cord clamping
PVR: pulmonary vascular resistance
VTI: velocity time integral
CC: correlation coefficient.
